# Submucosal hemorrhage of the esophagus: a case report

**DOI:** 10.1186/s44215-024-00175-1

**Published:** 2024-11-15

**Authors:** Risako Kojima, Shinsuke Takeno, Makoto Ikenoue, Teru Chiyotanda, Yusuke Araki, Kousei Tashiro, Fumiaki Kawano, Atsushi Nanashima, Kouji Furukawa

**Affiliations:** Department of Surgery, Faculty of Medicine, Miyazaki University, Miyazaki, 889-1692 Japan

**Keywords:** Submucosal hemorrhage of the esophagus, Esophageal hemorrhage, Epigastric pain, Conservative treatment

## Abstract

**Background:**

Submucosal hemorrhage of the esophagus is relatively rare and the course of this disease remains unclear. We report a case of this disease.

**Case presentation:**

The patient was a 68-year-old man who visited a clinic complaining of sudden-onset epigastric and back pain. He had been taking warfarin and a statin due to non-obstructive hypertrophic cardiomyopathy, right subclavian artery stenosis, and chronic atrial fibrillation. Contrast-enhanced computed tomography showed esophageal submucosal hemorrhage. Detailed endoscopic examination was difficult because of the massive hemorrhage and progressive esophageal mucosal edema. He was transferred to our hospital due to progression of anemia. Fortunately, hemorrhagic anemia showed no progression with conservative fasting therapy after admission to our hospital. Esophageal mucosa over the submucosal hemorrhage detached and regenerative tissue was observed on endoscopic examination 1 week later.

**Conclusions:**

Esophageal submucosal hemorrhage should be included among the differential diagnoses for patients presenting with chest and back pain.

## Background

Submucosal hemorrhage of the esophagus is rare and the course of this pathology is unclear. The causes are classified as idiopathic or traumatic. Idiopathic causes are considered to be due to increased esophageal pressure following overeating, vomiting, or coagulation abnormalities. Initial symptoms often include hematemesis, chest and back pain, epigastric pain, and difficulty swallowing [1]. This condition may develop with sudden chest pain, so differentiation from acute aortic dissection and myocardial infarction may be necessary [2]. Improvement is often seen with conservative treatment and the prognosis is good [3]. However, rare cases have reportedly required surgical treatment and treatment at a facility that can handle emergencies thus appears desirable [4]. We report a case of idiopathic submucosal hemorrhage of the esophagus in which a good outcome was achieved with conservative treatment.

## Case presentation

The patient was a 68-year-old man who had been taking warfarin and a statin due to non-obstructive hypertrophic cardiomyopathy, right subclavian artery stenosis, and chronic atrial fibrillation. He visited a local clinic with chief complaints of sudden-onset epigastric and back pain.

**Fig. 1:**
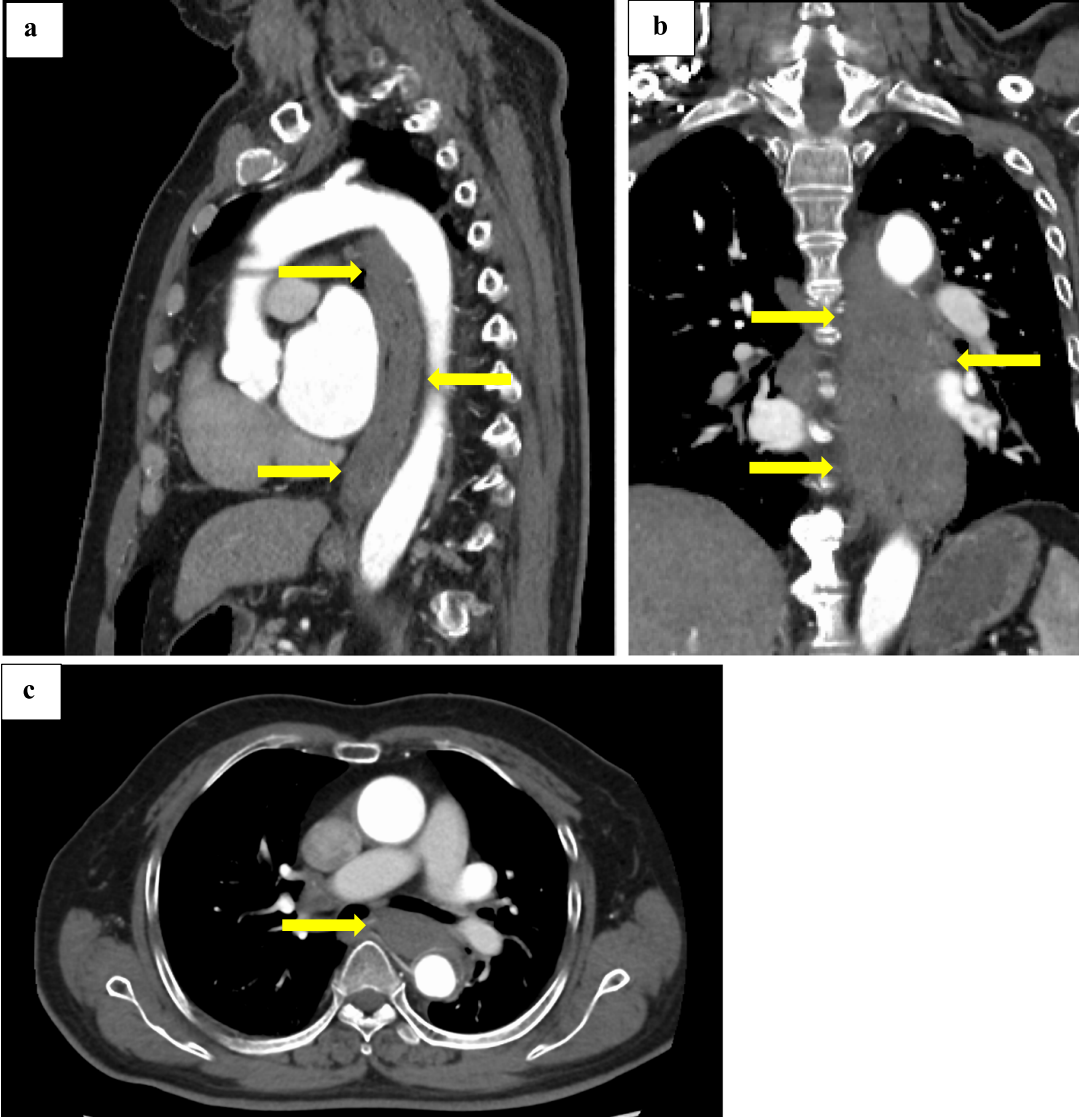
Contrast-enhanced CT at first visit. **a** Sagittal, **b** coronal, **c** axial. Severe edematous wall thickening with unclear contrast effect is evident throughout the esophagus

**Fig. 2:**
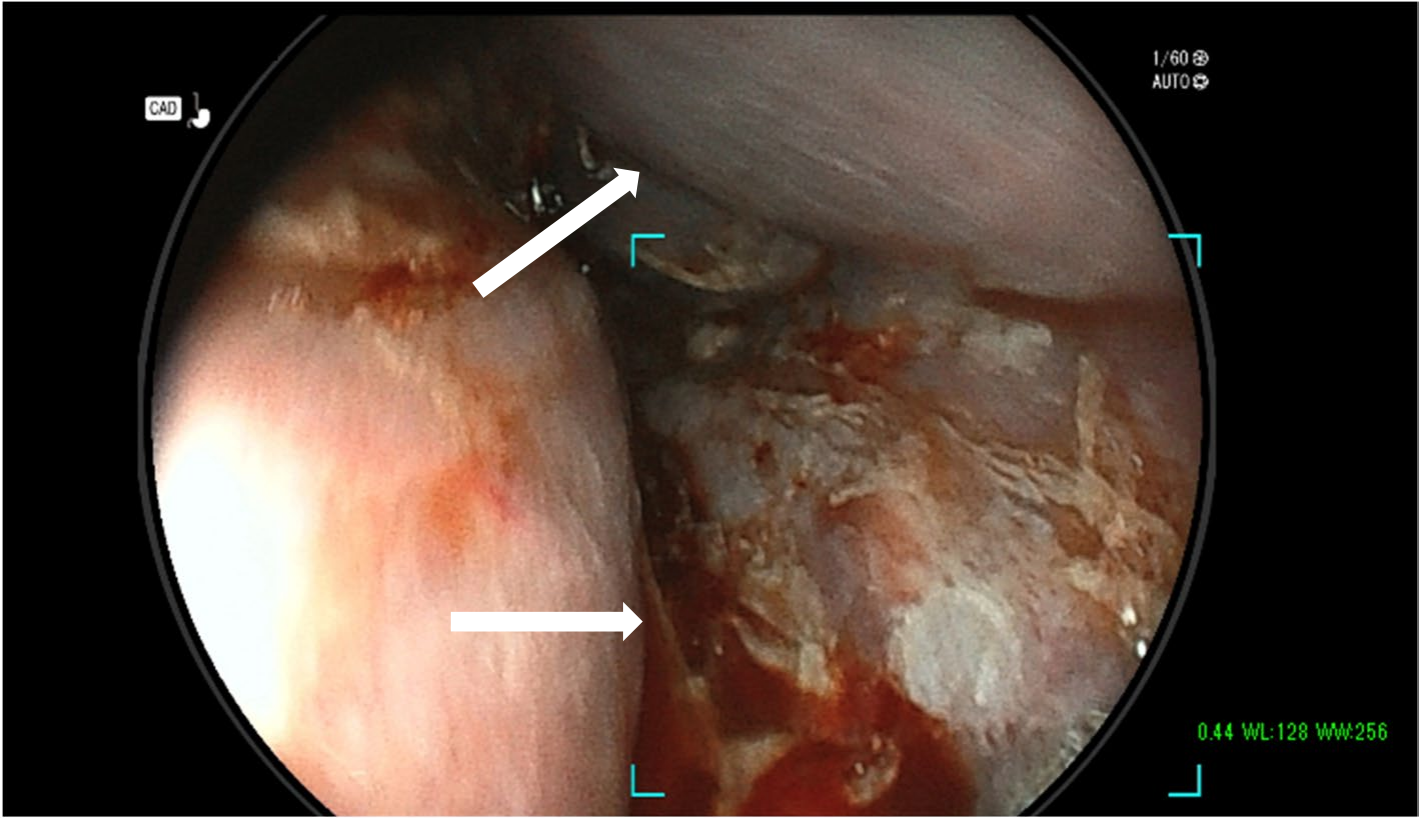
Upper gastrointestinal endoscopy on admission. Wall thickening and bleeding are seen in the middle of the esophagus. The mucosa shows ischemic coloration and severe edema is evident

Contrast-enhanced computed tomography (CT) showed severe edematous wall thickening with an unclear contrast effect throughout the esophagus (Fig. 1). Detailed endoscopic examination was difficult because of massive hemorrhage and progression of mucosal edema in the middle thoracic esophagus, but the esophageal mucosa showed ischemic changes (Fig. 2). He was admitted to a hospital for conservative treatment with fasting, withdrawal of warfarin, and administration of hemostatic agents under a diagnosis of esophageal submucosal hemorrhage.

**Fig. 3:**
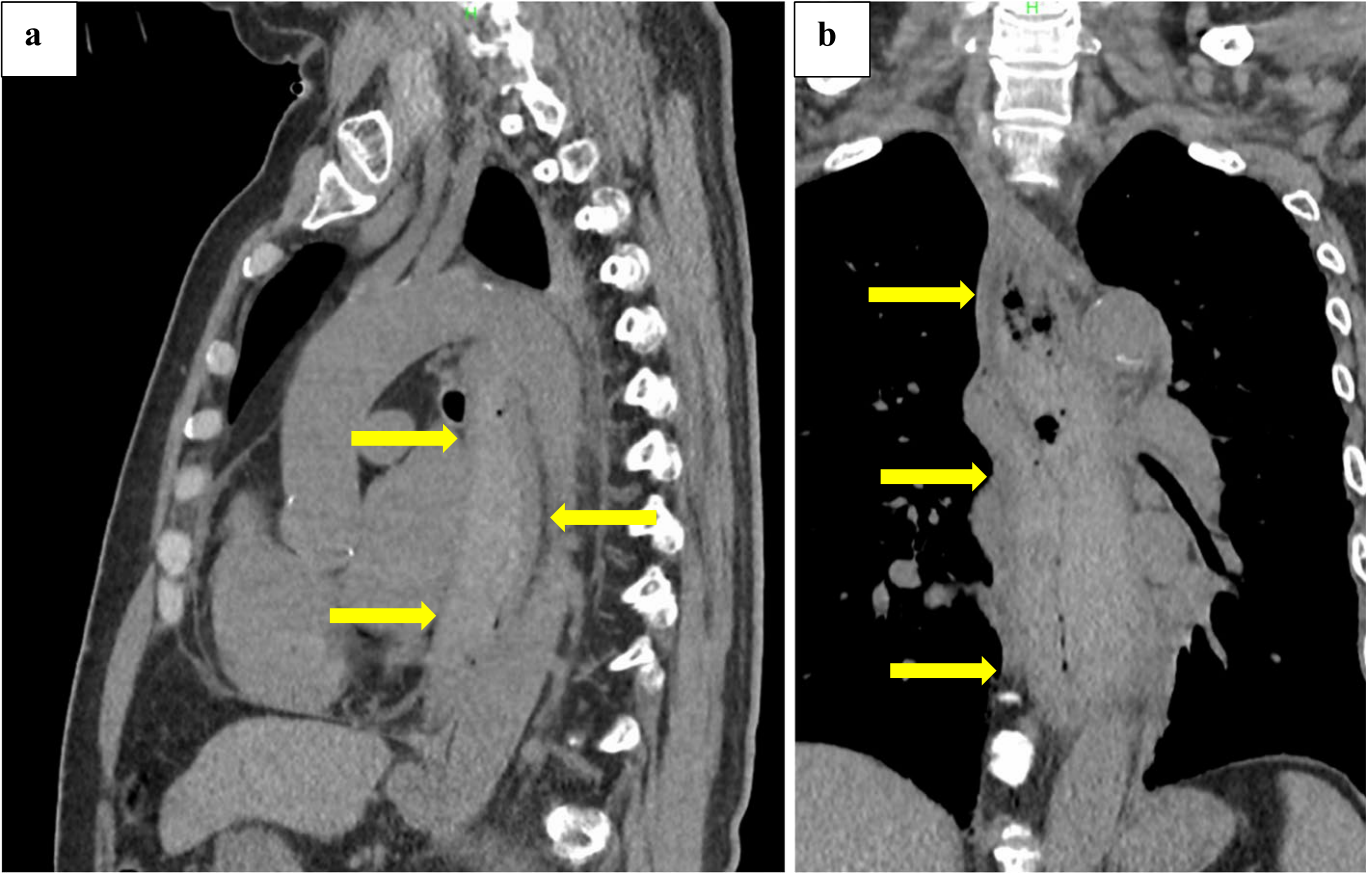
Plain CT on day 5 of illness. **a** Sagittal, **b** coronal. A high-density area due to hemorrhage is seen in the region of the wall thickening seen on CT on admission. Compared to the CT scan at the time of admission, it was thought that the bleeding was continuing

**Fig. 4:**
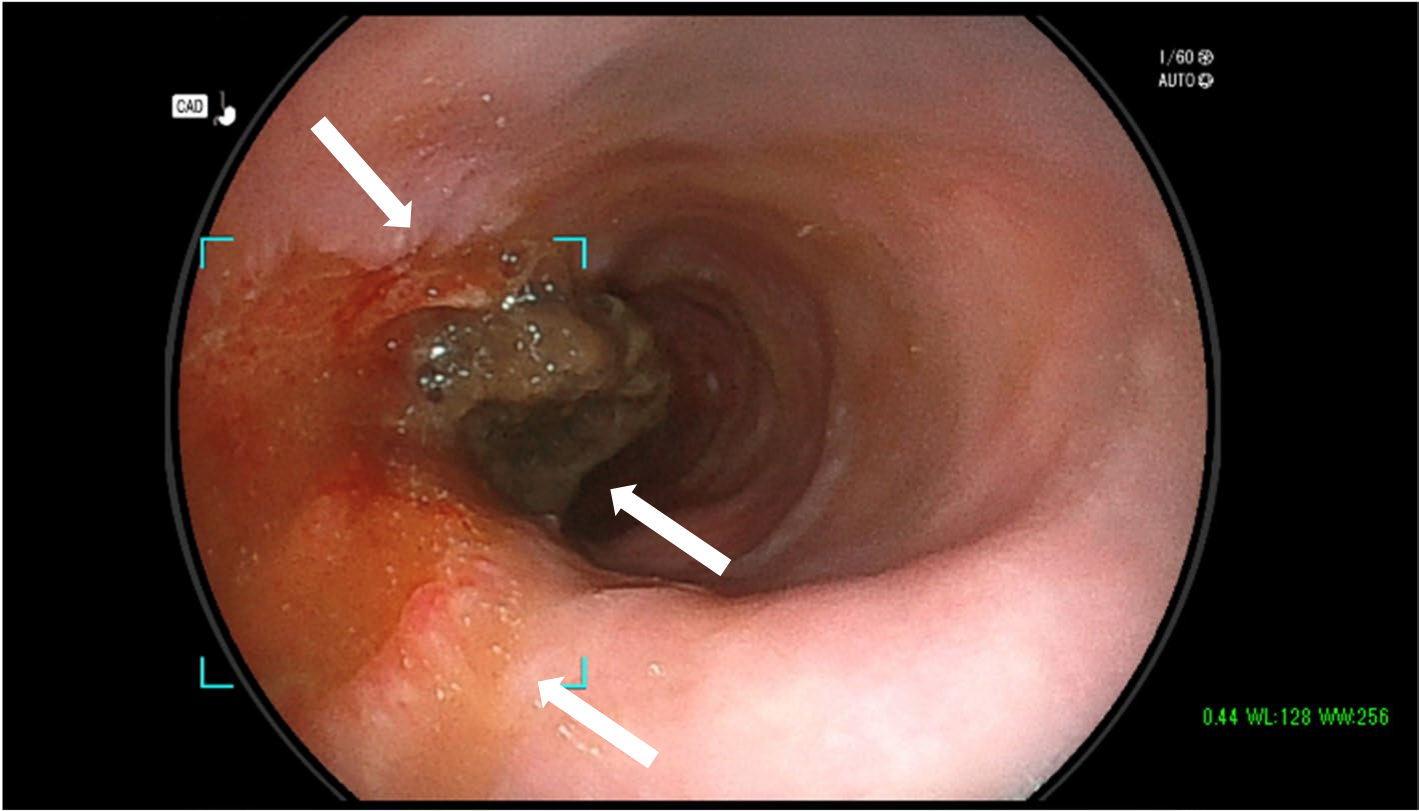
Endoscopy on day 6 of illness. Unilateral ulcers and strictures in the upper esophagus show easy contact bleeding

Plain CT on day 5 of illness showed a high-density area in the region of the wall thickening seen on CT on admission (Fig. 3). Endoscopy on day 6 revealed unilateral ulceration and stricture in the upper esophagus as well as contact oozing (Fig. 4). He was transferred to our hospital because of progression of anemia, for the purpose of urgent surgery on day 9 after onset.

**Fig. 5:**
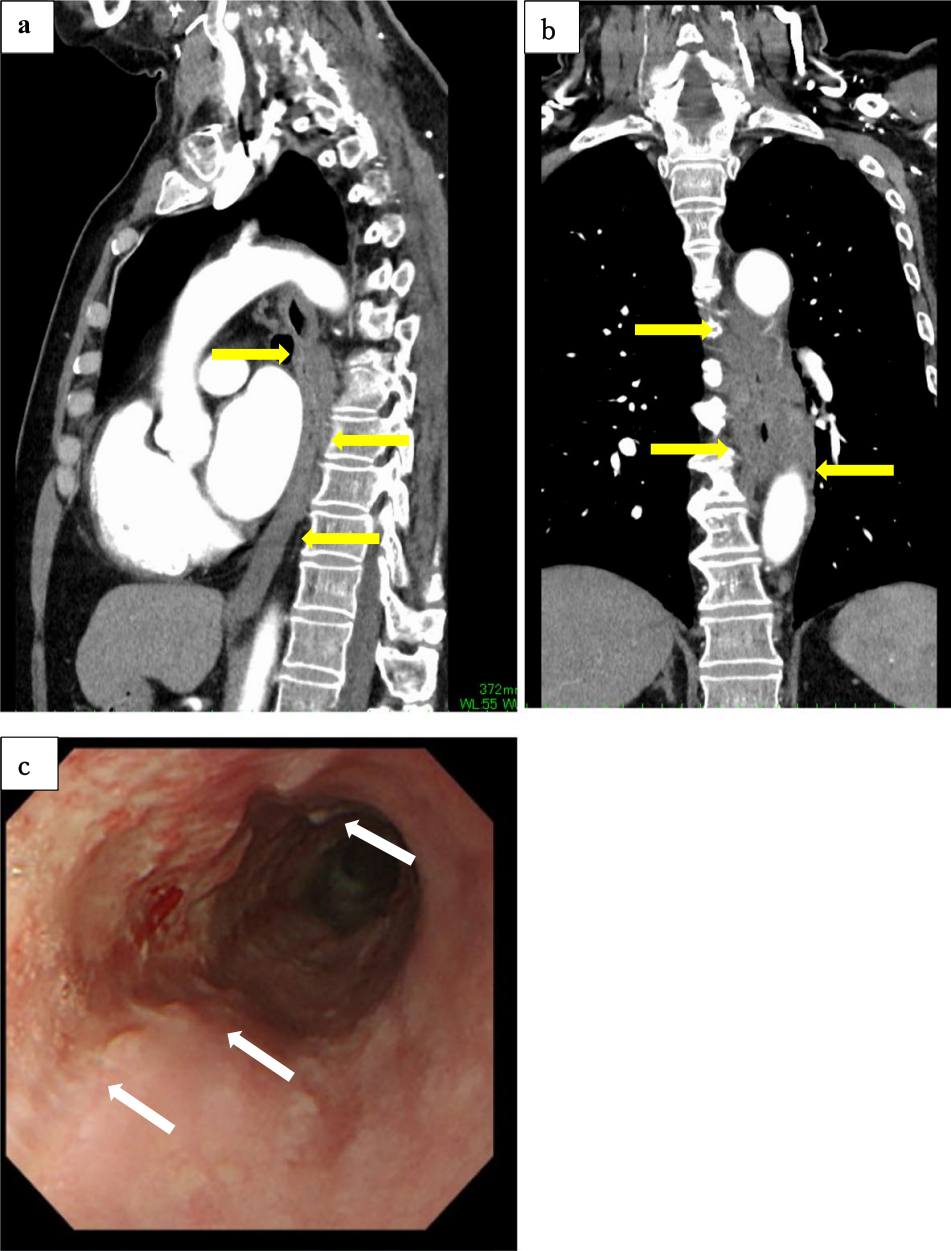
Investigations after transfer to our hospital. **a** Sagittal, **b** coronal. CT shows significant improvement in thickening of the esophageal wall. **c** Endoscopic observation of the ulcer and regenerated epithelium show improvement compared to the previous examination

Hemorrhagic anemia fortunately showed no progression with conservative fasting therapy after admission to our hospital. The esophageal mucosa over the submucosal hemorrhage detached and regenerative tissue was observed on endoscopic examination 1 week later (Fig. 5). The patient was discharged with an uneventful course after resumption of meals on day 19 of hospitalization.

## Discussion

Esophageal submucosal hemorrhage was first reported by Williams in 1957 as idiopathic esophageal submucosal dissection [5]. This pathology is due to blood vessel rupture in the esophageal submucosa, with causes divided into a traumatic type and an idiopathic type attributed to increases in esophageal pressure due to eating or vomiting [1]. Yamada et al. also reported that the insertion of a nasogastric tube may cause hematoma. [6] Oba et al. reported a case of Mallory–Weiss syndrome causing esophageal submucosal hematoma. [7] Initial symptoms often include hematemesis, chest and/or back pain, and difficulty swallowing. Endoscopy and enhanced esophagography are useful for diagnosis [8].

Almost all cases can be cured with conservative treatments such as fasting and the prognosis is favorable. However, some cases have been reported to require urgent surgery or interventional treatment. Shim et al. reported embolization of the esophageal branch of the left gastric artery with lipiodol for idiopathic intramural esophageal hematoma. [9] Isaac et al. reported that hemostasis was achieved by embolization of the mid-to-distal esophageal arterial branches from the thoracic aorta. Recovery with conservative therapy is considered difficult in cases with hemodynamic collapse or secondary to aortoesophageal fistula. [10] Some reports have suggested arterial embolization as a useful alternative to surgery [11].

The present case involved idiopathic esophageal submucosal hemorrhage that developed without any evident trigger. A favorable outcome was achieved by conservative treatment that included fasting and administration of hemostatic agents. We received a report of this patient several days after symptom onset, and he was transferred to another hospital because the anemia had progressed, but blood tests after that transfer showed that the anemia had stabilized. Conservative treatment reportedly improves the condition in most cases [1, 3, 4], so we decided on this approach.

Esophageal submucosal hemorrhage is reportedly more likely to occur among patients taking antithrombotic drugs or with underlying conditions creating a predisposition to bleeding [12]. In this case, the patient was taking warfarin. With the continued aging of populations worldwide, the number of patients taking antithrombotic drugs is increasing, and the chances of encountering this disease might be expected to increase in the future. Regular re-evaluation of new-onset chest pain and dysphagia/odynophagia during anticoagulation therapy and early recognition and management of esophageal submucosal hematomas may therefore prove beneficial [2].

In conclusion, esophageal submucosal hemorrhage should be included among the differential diagnoses for patients presenting with chest and back pain.

## Data Availability

Not applicable.

## Abbreviation

CT: Computed tomography

## References

[1] Tong M, Hung W-K, Law S, Wong K-H, Kwok K-F, Wong J. Esophageal hematoma. Dis Esophagus. 2006;19:200–2. 16722999 10.1111/j.1442-2050.2006.00565.x

[2] Hong M, Warum D, Karamanian A. Spontaneous intramural esophageal hematoma (IEH) secondary to anticoagulation and/or thrombolysis therapy in the setting of a pulmonary embolism: a case report. Radiology Case. 2013;7(2):1–10. 23705034 10.3941/jrcr.v7i2.1210 PMC3661306

[3] Folan RD, Smith RE, Head JM. Esophageal hematoma and tear requiring emergency surgical intervention: a case report and literature review. Dig Dis Sci. 1992;37(12):1918–21. 1473441 10.1007/BF01308089

[4] Furukawa H, Hara T, Taniguchi T, Tetsu O. A case of spontaneous intramural hematoma of the esophagus. Gastroenterol Jpn. 1993;28(1):81–7. 8440426 10.1007/BF02775007

[5] Williams B. Oesophageal laceration following remote trauma. Brit J Radiol. 1957;30:666–8. 13489209 10.1259/0007-1285-30-360-666

[6] Yamada T, Motomura Y, Hiraoka E, Miyagaki A, Sato J. Nasogastric tubes can cause intramural hematoma oh the esophagus. Am J Case Rep. 2019;20:224–7. 30783075 10.12659/AJCR.914133 PMC6394141

[7] Oba J, Usuda D, Tsuge S, Sakurai R, Kawai K, Matsubara S, et al. Hemorrhagic shock due to submucosal esophageal hematoma along with Mallory-Weiss syndrome: a case report. World J Clin Cases. 2022;10(27):9911–20. 36186194 10.12998/wjcc.v10.i27.9911 PMC9516938

[8] Oe S, Watanabe T, Kume K, Shibata M, Hiura M, Yoshikawa I, et al. A case of idiopathic gastroesophageal submucosal hematoma and its disappearance observed by endoscopy. J UOEH. 2014;36(2):123–8. 24930876 10.7888/juoeh.36.123

[9] Jaejun Shim, Jae Young Jang, Young Hwangbo, Seok Ho Dong, Joo Hyeong Oh, Hyo Jong Kim, *et al.* Recurrent massive bleeding due to dissecting intramural hematoma of the esophagus: treatment with therapeutic angiography. *World J Gastroenterol* 2009; 15(41): 5232–5235 10.3748/wjg.15.5232 PMC2773907 19891027

[10] Mina F.G. Isaac, Chi Long Ho, Sum Leong. Life-threatening bleeding from dissecting intramural hematoma of esophagus (IHE) treated by trans arterial embolization. *Radiology Case Reports* 2021; 16: 2474–2477 10.1016/j.radcr.2021.05.067 PMC8260735 34257783

[11] Gao F, Zhang T, Guo X, Zhe Su. Embolization of the esophageal branch of intercostal artery for treatment of spontaneous intramural hematoma of the esophagus: a case description. Quant Imaging Med Surg. 2023;13(10):7417–22. 37869337 10.21037/qims-23-564 PMC10585536

[12] E. Cummins, M. Sharkey, T. Eastin, E. Adkins. A rare cause for acute chest pain in the emergency setting that is hard to swallow. *Case Reports in Emergency Medicine* 2013, Article ID 646342, 4 pages 10.1155/2013/646342 PMC3730207 23956888

